# Anti-Cancer Effect of Quercetin in Xenograft Models with EBV-Associated Human Gastric Carcinoma

**DOI:** 10.3390/molecules21101286

**Published:** 2016-09-26

**Authors:** Hwan Hee Lee, Seulki Lee, Yu Su Shin, Miyeon Cho, Hyojeung Kang, Hyosun Cho

**Affiliations:** 1 College of Pharmacy, Duksung Women’s University, Seoul 132-714, Korea; oeo3oeo@gmail.com (H.H.L.); seulki0614@duksung.ac.kr (S.L.); 2 Innovative Drug Center, Duksung Women’s University, Seoul 132-714, Korea; 3 Department of Medicinal Crop Research, National Institute of Horticultural and Herbal Science, Rural Development Administration, Eumseong 369-873, Korea; totoro69@korea.kr; 4 College of Pharmacy, Research Institute of Pharmaceutical Sciences and Institute for Microorganisms, Kyungpook National University, Daegu 702-701, Korea; cmy1004g@naver.com

**Keywords:** EBV, human gastric carcinoma, p53, quercetin, SNU719

## Abstract

Licorice extracts have been widely used in herbal and folk medications. *Glycyrrhiza* contains diverse range of biological compounds including triterpenes (glycyrrhizin, glycyrrhizic acid) and flavonoids (quercetin, liquiritin, liquiritigenin, glabridin, licoricidin, isoliquiritigenin). The flavonoids in licorice are known to have strong anti-cancer activities. Quercetin, the most abundant flavonoid, has been shown to have anti-ulcer, anti-cancer, antioxidant, and anti-inflammatory properties. Latent Epstein-Barr virus (EBV) infection can lead to serious malignancies, such as, Burkitt’s lymphoma, Hodgkin’s disease and gastric carcinoma(GC), and (Epstein-Barr virus associated gastric carcinoma) EBVaGC is one of the most common EBV-associated cancers. In this study, the authors first examined the anti-cancer effects of quercetin and isoliquiritigenin in vivo xenograft animal models implanted with EBV(+) human gastric carcinoma (SNU719) or EBV(−) human gastric carcinoma (MKN74), and then explored the molecular mechanisms responsible for their anti-cancer activities. The results obtained showed that anti-cancer effect of quercetin was greater than isoliquiritigenin in mice injected with EBV(+) human gastric carcinoma (SNU719) cells. On the other hand, quercetin and isoliquiritigenin had similar anti-cancer effects in mice injected with EBV(−) human gastric carcinoma (MKN74) cells. Interestingly, quercetin inhibited EBV viral protein expressions, including EBNA-1 and LMP-2 proteins in tumor tissues from mice injected with EBV(+) human gastric carcinoma. Quercetin more effectively induced p53-dependent apoptosis than isoliquiritigenin in EBV(+) human gastric carcinoma, and this induction was correlated with increased expressions of the cleaved forms of caspase-3, -9, and Parp. In EBV(−)human gastric carcinoma (MKN74), both quercetin and isoliquiritigenin induced the expressions of p53, Bax, and Puma and the cleaved forms of caspase-3 and -9 and Parp at similar levels.

## 1. Introduction

Licorice is the root of *Glycyrrhiza glabra* or *Glycyrrhiza uralensis*, a perennial legume found in southern Europe (*Glycyrrhiza glabra)* and in east Asia (*Glycyrrhiza uralensis*). Licorice extracts have been widely used in herbal and folk medications. *Glycyrrhiza* contains a diverse range of biological compounds including triterpenes (glycyrrhizin, glycyrrhizic acid) and the flavonoids (quercetin, liquiritin, liquiritigenin, glabridin, licoricidin, isoliquiritigenin) [1].

The flavonoids of licorice include quercetin, isoliquiritigenin, and liquiritin, and all three have been shown to have strong anti-cancer activity [2]. Isoliquiritigenin has been reported to stimulate cell cycle arrest in human prostate cancer cells and to induce the death of human breast cancer and gastric cancer cells [3,4,5]. Isoliquiritigenin was found to suppress human lung cancer cell growth and colon cancer in mice [6]. These observations suggest isoliquiritigenin has great potential for cancer prevention and therapy. The anti-viral effects of isoliquiritigenin have only been reported by a few studies. Sekine-Osajima et al. and Adianti et al. found isoliquiritigenin strongly inhibits Hepatitis C virus (HCV) replication [7,8].

Quercetin, the most abundant compound in flavonoids, has been shown to possess anti-ulcer, anti-cancer, antioxidant, and anti-inflammatory properties [9,10,11]. Furthermore, it has been shown to inhibit the growth of various cancer cells by inducing apoptosis [12,13], and to have a protective effect in animal models of colon cancer [14]. In addition, two studies have reported quercetin prevented gastric ulcer induced mucosal damage [15,16], and interestingly, the anti-ulcer effect of quercetin was found to be associated with the inhibition of *Helicobacter pylori* growth [17]. Recently, quercetin was reported to have a robust anti-viral activity. It inhibited HBsAg and HBeAg secretion in HBV producing cells and HCV replication in a HCV replicon culture system [18,19]. Quercetin and its structural analogs were also reported to suppress HIV-1 reverse transcriptase and other reverse transcriptases from avian myeloblastosis, Rous-associated virus-2, and Maloney murine leukemia virus [20,21].

Epstein-Barr virus (EBV) is a human gamma-1 herpesvirus with lifetime latency and infects over 90% of the world’s population [22]. EBV survives mainly in a chromatin-associated, multicopy episome in the nuclei of various transformed tumor cells derived from B cells, T cells, and epithelial cells. Latent EBV infection can lead to serious malignancies, such as, Burkitt’s lymphoma, Hodgkin’s disease, and gastric carcinoma [23,24]. In fact, approximately 10% of GCs are EBVaGC [25], which is known to be one of the most common EBV-associated tumors [26].

In our previous study, we investigated the antitumor effects of quercetin and isoliquiritigenin against EBVaGC, and their antiviral effects against EBV. We found both quercetin and isoliquiritigenin were cytotoxic to EBVaGC SNU719 cells, and that quercetin induced more apoptosis and cell cycle arrest than isoliquiritigenin in SNU719 cells. Furthermore, whereas quercetin reduced EBV latency and inhibited EBV infection, isoliquiritigenin increased EBV latency. These results suggest that quercetin could be a promising lead compound for EBV associated gastric carcinoma [27].

In this study, we examined the anti-cancer effects of quercetin and isoliquiritigenin in in vivo xenograft models implanted with EBV(+) human gastric carcinoma (SNU719) or EBV(−) human gastric carcinoma (MKN74). In addition, we explored the molecular mechanisms responsible for the anti-cancer activities of quercetin and isoliquiritigenin using tumor tissues derived from in vivo xenograft animals implanted with EBV(+) human gastric carcinoma (SNU719) or EBV(−) human gastric carcinoma (MKN74).

## 2. Results and Discussion

### 2.1. Anti-Tumor Effects of Quercetin and Isoliquiritigenin in Xenograft NOD/SCID Mice Bearing EBV(+) or EBV(−) Human Gastric Carcinoma (SNU719 or MKN74)

**Figure 1:**
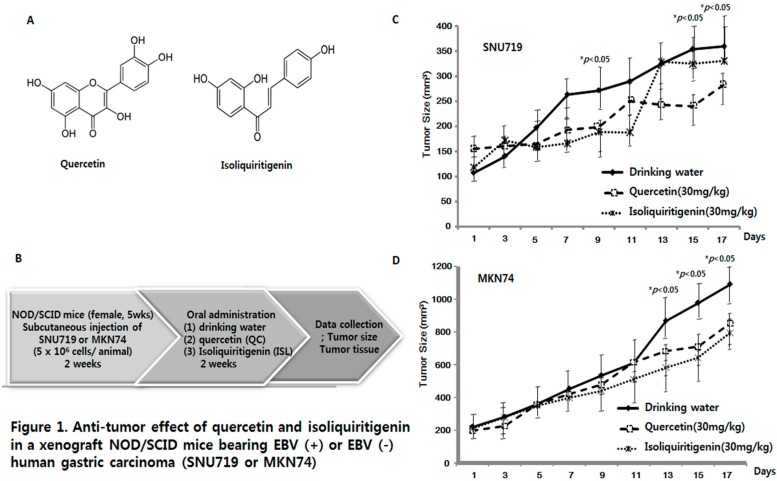
Anti-tumor effects of quercetin and isoliquiritigenin in xenograft NOD/SCID mice bearing EBV(+) or EBV(−) human gastric carcinoma (SNU719 or MKN74) NOD/SCID mice were randomly divided into two groups. One group was implanted with EBV(+) human gastric carcinoma, SNU719, and the other group was implanted with EBV(−) human gastric carcinoma, MKN74. These two groups were then subdivided into three subgroups (*n* = 5/subgroup). Two weeks after implantation, mice were orally administrated (in drinking water) quercetin (QC, 30 mg/kg/day) or isoliquiritigenin (ISL, 30 mg/kg/day). (**A**) The structures of quercetin and isoliquiritigenin; (**B**) The study design of the animal experiment; (**C**) Tumor sizes in animals injected with EBV(+) positive carcinoma (SNU719) or (**D**) EBV(−) carcinoma (MKN74).

To evaluate the anti-tumor effects of quercetin (QC) and isoliquiritigenin (ISL) in vivo, 30 xenograft mice were randomly divided into two groups and subcutaneously injected with EBV(+) or EBV(−) cells (5 × 10^6^ cells per mouse), SNU719, or MKN74, respectively. Two weeks later, each group was randomly divided into three subgroups, which administered drinking water (DW), quercetin (30 mg/kg/day), or isoliquiritigenin (30 mg/kg/day) orally for two weeks. Tumor size was measured every other day. Figure 1A displays the structures of quercetin and isoliquiritigenin and Figure 1B shows the overall design of the animal experiment. Figure 1C shows that quercetin (QC) or isoliquiritigenin (ISL) inhibited the growth of EBV(+) human gastric carcinoma (SNU719) from day 5 (DW, 197.3 mm^3^; QC, 166.0 mm^3^; ISL, 159.1 mm^3^) through day 11 (DW, 290.6 mm^3^; QC, 250.9 mm^3^; ISL, 188.4 mm^3^), and the growth inhibition was significant on day 9 (DW, 271.9 mm^3^; QC, 199.5 mm^3^; ISL, 189.4 mm^3^). Interestingly, quercetin continued to inhibit the tumor growth of EBV(+) human gastric carcinoma (SNU719) whereas isoliquiritigenin did not from day 13 (DW, 325.5 mm^3^; QC, 243.8 mm^3^; ISL, 328.8 mm^3^) until the study end point (day 21) (DW, 360.2 mm^3^; QC, 283.8 mm^3^; ISL, 330.8 mm^3^). In case of EBV(−) human gastric carcinoma (MKN74) (Figure 1D), quercetin and isoliquiritigenin exhibited delayed tumor inhibitory effects, but in EBV(−) human gastric carcinoma (MKN74) inhibited growth from day 13 (DW, 868.9 mm^3^; QC, 683.7 mm^3^; ISL, 581.9 mm^3^) until day 17 (DW, 1093.0 mm^3^; QC, 854.5 mm^3^; ISL, 795.8 mm^3^). These results indicate quercetin and isoliquiritigenin have anti-tumor effects on human gastric carcinoma, but that quercetin has a greater effect on the EBV-associated gastric carcinoma (SNU719).

In fact, many studies have shown quercetin and isoliquiritigenin have inhibitory effects on various human cancer cell lines, such as, ovarian, breast, and bladder cancer cells in vitro [13,28,29]. A few groups have reported quercetin has an inhibitory effect on xenograft mouse bearing human prostate carcinoma PC-3 [30,31] or human breast cancer (MCF-7) cells [32]. The anti-tumor effect of isoliquiritigenin has been examined in xenograft mouse bearing human lung cancer [33], but the anti-cancer effects of quercetin and isoliquiritigenin on human gastric carcinoma have not been previously examined in xenograft animal models. Therefore, this is the first study to evaluate the anti-cancer effects of quercetin and isoliquiritigenin using in vivo xenograft animal models bearing human gastric carcinoma, especially in presence or absence of EBV. The in vivo anti-cancer effect of quercetin and isoliquiritigenin certainly correlates with the in vitro inhibitory effect on SNU719 cells that was reported in our previous study (Figure 1C,D) [34].

### 2.2. Down-Regulations of EBV EBNA1 and LMP-2 in Tumor Tissues from Mice Implanted with EBV(+) Human Gastric Carcinoma (SNU719)

**Figure 2:**
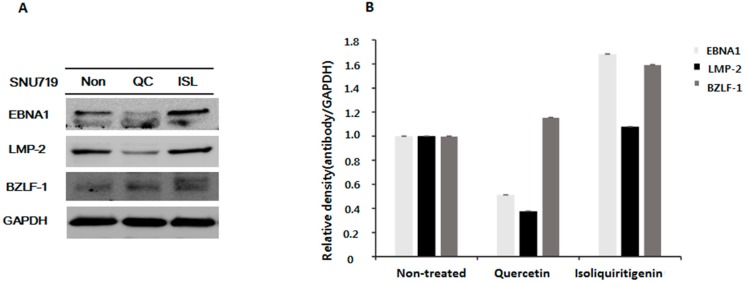
Expressions of EBNA1, LMP-2, and BZLF-1 proteins in tumor tissues from mice implanted with EBV(+) human gastric carcinoma (SNU719) EBV(+) human gastric carcinoma tumor tissue was excised from each animal fed quercetin (QC) or isoliquiritigenin (ISL) and prepared for western blot analysis. (**A**) The protein expression of EBNA1, LMP-2, and BZLF-1 were identified and (**B**) relative intensities were measured. GAPDH was used as the loading control.

Surprisingly, the anti-cancer effect of quercetin was retained through the study, but the anti-cancer effect of isoliquiritigenin disappeared on day 13 in EBV(+) human gastric carcinoma (Figure 1C). We speculated that the anti-cancer mechanism of isoliquiritigenin might be differentially regulated in the presence of EBV. Of note, we previously suggested quercetin has a greater anti-viral effect than isoliquiritigenin because it affects EBV replication [27]. Therefore, we analyzed the expression of EBV proteins (EBNA1, LMP-2, and BZLF-1) in EBV(+) tumor tissues derived from quercetin fed mice and compared these with EBV(+) tumor tissues derived from isoliquiritigenin fed animals by performing Western blot assays using anti-EBV EBNA1, LMP-2, and BZLF-1 antibodies (Figure 2). EBV EBNA1 and LMP-2 proteins are known to be essential for EBV latency and EBV BZLF-1 is a key factor for EBV lytic reactivation [27]. As shown in Figure 2A,B, the expressions of EBNA1 and LMP-2 were highly suppressed by quercetin, but not by isoliquiritigenin, suggesting quercetin has a stronger anti-EBV effect than isoliquiritigenin. In fact, in a previous study, we found EBV infection of gastric adenocarcinoma cells was severely inhibited by quercetin but unaffected by isoliquiritigenin [27]. Therefore, we speculate that the anti-viral effect of quercetin on EBV infection might contribute in some way to its anti-cancer effect in EBV(+) human gastric carcinoma because EBV is known to cause and promote gastric carcinoma.

### 2.3. Increased Expressions of p53, p21, and of Apoptotic Proteins in Tumor Tissues from Mice Implanted with EBV(+) Human Gastric Carcinoma (SNU719)

The anti-tumor mechanisms of quercetin or isoliquiritigenin have been intensively examined in vitro. In particular, in human gastric carcinoma, quercetin and isoliquiritigenin were reported to induce apoptosis or cell cycle arrest in MGC-803 cells and SNU719 cells [5,27,34].

The tumor suppressive protein p53 plays an important role in cell apoptosis in response to DNA damage. It also regulates the expression of PUMA, which interacts with antiapoptotic Bcl-2 family members. Their interaction makes Bax free, which signals apoptosis to mitochondria and leads to caspase activation and cell death [35]. Therefore, the functions of p53 and its associated proteins, such as, p21, Bax, and PUMA, are important in cancer studies [35].

**Figure 3:**
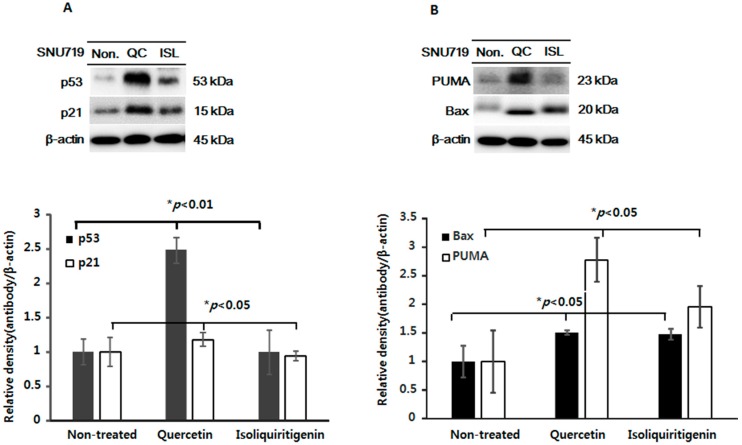
Expression of p53, p21, PUMA, and Bax in tumor tissues from mice implanted with EBV(+) human gastric carcinoma (SNU719) EBV(+) human gastric carcinoma tumor tissue was excised from each animal fed quercetin (QC) or isoliquiritigenin (ISL) and prepared for western blot analysis. The protein expressions of (**A**) p53, p21; (**B**) PUMA and Bax were identified and the relative intensities were measured. β-Actin was used as the loading control.

We examined the effect of quercetin or isoliquiritigenin on cell apoptosis as well as cell cycle regulation in EBV(+) human gastric carcinoma (SNU719) using flow cytometry. Both quercetin and isoliquiritigenin induced a significant cell apoptosis, but they had little effect on the cell cycle (Supplementary Materials Figures S1 and S2), which partly correlates with our previous study [27]. Next, we investigated the molecular mechanisms initiated by quercetin or isoliquiritigenin in EBV(+) human gastric carcinoma (SNU719). Tumor tissues were harvested from animals bearing EBV(+) human gastric carcinoma and then lysed using buffer solution. Levels of p53, p21, Bax, and PUMA were then assessed in lysate proteins. We found increased expression of p53 and p21 in EBV(+) human gastric carcinoma bearing animals fed quercetin (Figure 3A), which achieved a statistically significant increase, indicating quercetin mediates its anti-tumor effect in a p53 and p21 dependent manner in EBV(+) human gastric carcinoma. In case of isoliquiritigenin, it slightly increased the expression of p53, but not p21, which was not statistically significant. On the other hand, both quercetin and isoliquiritigenin significantly augmented the expressions of Bax and PUMA in EBV(+) human gastric carcinoma (Figure 3B).

### 2.4. Increased Expressions of Cleaved Caspase-3 and -9 and Cleaved Parp Proteins in Tumor Tissues from Nice Implanted with EBV(+) Human Gastric Carcinoma (SNU719)

**Figure 4:**
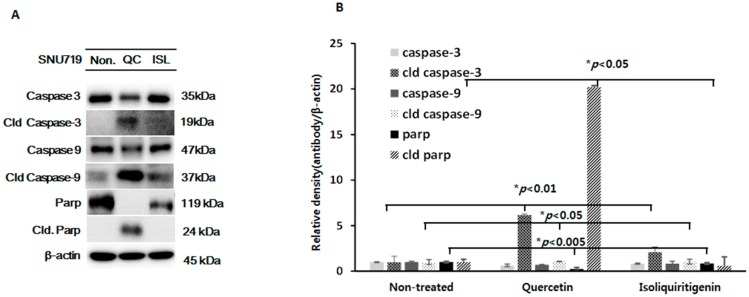
Expressions of (cleaved) caspase-3, -9, and (cleaved) Parp proteins in tumor tissues from mice implanted with EBV(+) human gastric carcinoma (SNU719) EBV(+) human gastric carcinoma tumor tissue was excised from each animal fed quercetin (QC) or isoliquiritigenin (ISL) and prepared for western blot analysis. (**A**) The expressions (cleaved) caspase-3, -9, and (cleaved) Parp were identified and (**B**) relative intensities were measured. β-Actin was used as the loading control.

Mitochondria-mediated apoptosis involves not only p53-related signaling molecules but also the activations of caspases and poly (ADP-ribose) polymerase (Parp) [36]. Caspases are well-known proteases and are classified as initiators (caspase-2, -8, -9, and -10) or effectors (caspase-3, -6, and -7). Caspases initiate two different apoptotic pathways, the extrinsic and intrinsic pathways. Apoptosis through the intrinsic pathway is triggered by endogenous stimuli, such as, DNA damage and oxidative stress [37]. Recently, Seo et al. reported quercetin induced cell apoptosis in a caspase-dependent manner in HER-2 overexpressing BT-474 breast cancer cells [38] and that isoliquiritigenin increased the expressions of caspase-3, Parp, and Bim in human lung adenocarcinoma [33]. In the present study, both quercetin and isoliquiritigenin lead to the cleavage of caspase-3 and -9 in tumor tissues derived from EBV(+) human gastric carcinoma (Figure 4). Of note, quercetin increased the expressions of the cleaved forms of caspase-3 and -9 more than isoliquiritigenin (Figure 4A,B). The expressions of cleaved Parp proteins were also obviously upregulated in EBV(+) human gastric carcinoma bearing animals fed quercetin, but not in EBV(+) human gastric carcinoma bearing animals fed isoliquiritigenin (Figure 4A). We consider that the phenotypically different effects of quercetin and isoliquiritigenin on the expressions of the cleavage forms of caspase-3, -9, and Parp probably contribute to their differential anti-cancer effects (Figure 1B). In addition, we suggest the stronger effect of quercetin on cell death-related molecules (caspases and Parp) in EBV(+) human gastric carcinoma might be influenced by its anti-EBV activity (Figure 2A,B).

### 2.5. Increased Expressions of p53, Bax, PUMA, Cleaved Caspase-3 and -9, and Cleaved Parp Proteins in Tumor Tissue from Mice Implanted with EBV(−) Human Gastric Carcinoma (MKN74) 

We also examined the effect of quercetin or isoliquiritigenin on cell apoptosis and cell cycle arrest in EBV(−) human gastric carcinoma (MKN74). Quercetin induced a significant cell apoptosis in a dose dependent manner (Supplementary Materials Figure S3). In case of cell cycle regulation, only isoliquiritigenin had a slight effect on cell cycle arrest (Supplementary Materials Figure S4).

**Figure 5:**
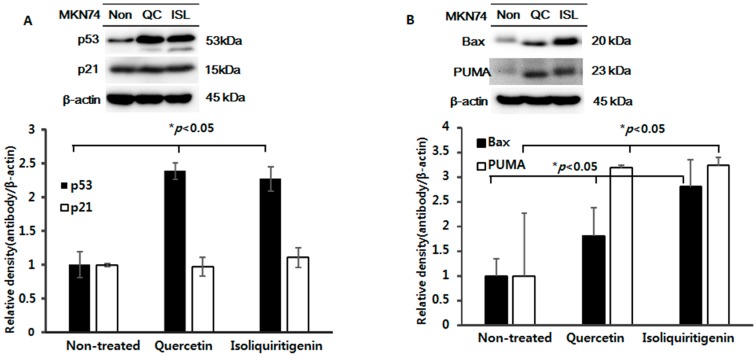

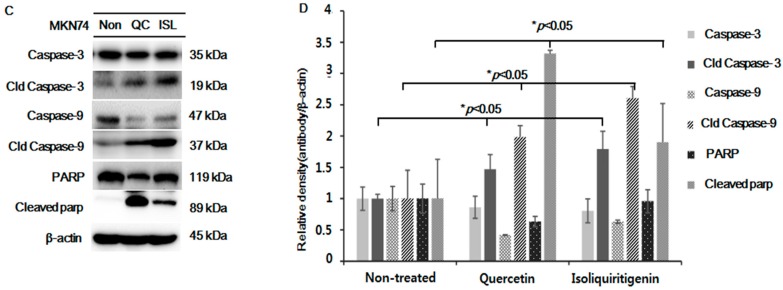
Expressions of p53, p21, Bax, PUMA, (cleaved) caspase-3, -9 and (cleaved) Parp proteins in tumor tissues from mice implanted with EBV(−) human gastric carcinoma (MKN74) EBV(−) tumor tissue was excised from each animal and prepared for western blot analysis. The protein expressions of (**A**) p53, p21; (**B**) Bax, PUMA; (**C**,**D**) (Cleaved) caspase-3, -9, and Parp were identified and relative intensities were measured. β-Actin was used as the loading control.

We investigated the anti-tumor mechanism of quercetin or isoliquiritigenin in tumor tissues from mice implanted with EBV(−) human gastric carcinoma (MKN74). It was found quercetin and isoliquiritigenin increased the expressions of p53, Bax, and PUMA, but not that of p21 (Figure 5A,B). These results suggest that quercetin and isoliquiritigenin induce p53-dependent apoptosis in EBV(−) human gastric carcinoma (MKN74), which is a little different from the result shown in Figure 3A for EBV(+) human gastric carcinoma (SNU719). Accordingly, we speculate that tumorigenesis was regulated in different ways in these cells.

We also examined whether the expressions of caspases or Parp were changed in EBV(−) human gastric carcinoma (MKN74). As was expected, quercetin and isoliquiritigenin both markedly upregulated the cleaved forms of caspase-3, -9, and Parp (Figure 5C,D). Interestingly, this upregulation of cleaved Parp by isoliquiritigenin was not observed in EBV(+) human gastric carcinoma (SNU719). These results match with the anti-cancer effects shown in Figure 1D, and suggest that quercetin and isoliquiritigenin provide similar levels of anti-tumor activity in EBV(−) human gastric carcinoma (MKN74).

## 3. Materials and Methods

### 3.1. Specimen Preparation and Cell Culture

Quercetin and isoliquiritigenin were purchased from Sigma-Aldrich Co. (St. Louis, MO, USA), and, before use, were dissolved in 10% DMSO (Sigma) and filtered through 0.22-μm filter. Human gastric carcinoma EBV(+) SNU719 and EBV(−) MKN74 cells were cultured in RPMI (Gibco, Life Technologies, Carlsbad, CA, USA) supplemented with 10% heat-inactivated fetal bovine serum (Hyclone, GE Healthcare Life Sciences, Logan, UT, USA), 100 U/mL penicillin/streptomycin (Gibco) at 37 °C in a humidified 5% CO_2_/95% air atmosphere.

### 3.2. In Vivo Experiments Using a Xenograft NOD/SCID Model

Animal experiments were conducted in accordance with the National Research Council’s Guide (IACUC, Seoul, Korea) for the Care and Use of Laboratory Animals. The experimental protocol was approved by the Animal Experiments Committee of Duksung Women’s University (permit number: 2014-016-007). NOD/SCID mice (female, five weeks old; Raonbio Co., Ltd., Seoul, Korea) were used as xenograft animal models. Mice were individually accommodated in a pathogen free controlled environment (23–27 °C under a 12-h day/12-h night cycle) and provided food and water ad lib. To produce tumors, mice were first divided into two groups (*n* = 15). The animals in one group were subcutaneously implanted with 5 × 10^6^ cells of EBV(+) human gastric carcinoma, SNU719 cells into the dorsum next to the right hind leg. The animals in the other group were implanted with 5 × 10^6^ cells of EBV(−) human gastric carcinoma, MKN74 cells in the same manner. After 14 days, both groups were subdivided into three subgroups (*n* = 5) and orally administrated drinking water, quercetin (30 mg/kg), or isoliquiritigenin (30 mg/kg) for two weeks. Tumors were identified and measured every other day using a standard caliper; tumor size was calculated using [tumor length (mm) × tumor width (mm)^2^]/2 as previously described [39,40]. After tumor size had reached 2000 mm^3^, animals were euthanized and tumors were harvested.

### 3.3. Western Blot Analysis

Tumor tissue specimens were harvested from EBV(+) and EBV(−) human gastric carcinoma SNU719 or MKN74 bearing mice and lysed using buffer solution (PRO-PREP Protein Extraction Solution, Intron, Korea). Protein level in tumor tissue lysates was measured using the Bradford Assay Plus Kit (Gen DEPOT. Inc., Baker, TX, USA), separated by electrophoresis, and transferred to nitrocellulose membranes. Membranes were incubated with first and second antibodies and blots were detected using enhanced chemiluminescent (ECL) reagents (GE Healthcare Life Sciences, Logan, UT, USA). The primary antibodies used were anti-p53 (clone BP53-12, Millipore, Billerica, MA, USA), anti-p21, anti-Bax (Cell signaling, Danvers, MA, USA), Apoptosis Sampler Kit (#9915, Cell signaling), and anti-β-actin Ab (Sigma Aldrich) [39,40].

### 3.4. Statistical Analysis

The significances of intergroup difference were determined by ANOVA. *p* values of less than 0.05 were considered statistically significant. 

## 4. Conclusions

In this study, we assessed the anti-cancer effects of quercetin and isoliquiritigenin using in vivo xenograft animals implanted with EBV(+) or EBV(−) human gastric carcinoma. Both quercetin and isoliquiritigenin exhibited anti-cancer effects on EBV(+) and EBV(−) human gastric carcinoma. However quercetin had the greater effect on the EBV(+) gastric carcinoma, which appears to be in line with anti-EBV effect of quercetin.

In addition, the protein expressions of p53, p21, Bax, and PUMA were elevated in EBV(+) human gastric carcinoma bearing animals fed quercetin or isoliquiritigenin, but only quercetin did so significantly. Quercetin also increased the expressions of the cleaved forms of caspase-3, -9, and Parp in EBV(+) human gastric carcinoma markedly more than isoliquiritigenin. However, both quercetin and isoliquiritigenin markedly upregulated the cleaved forms of caspase-3, -9, and Parp in EBV(−) human gastric carcinoma (MKN74), which matched their anti-cancer effects in tumor cells without EBV. We conclude that quercetin is a good candidate anti-cancer agent in EBV-associated human gastric carcinoma.

## Supplementary Materials

Supplementary materials can be accessed at: http://www.mdpi.com/1420-3049/21/10/1286/s1.
