# Supplementary material for: Anti-Cancer Effect of Quercetin in Xenograft Models with EBV-Associated Human Gastric Carcinoma
Source: Molecules. 2016 Sep 26;21(10):1286. doi: 10.3390/molecules21101286 (PMC6274130; doi:10.3390/molecules21101286)
Supplement: Supplementary file 1 [file molecules-21-01286-s001.pdf]

Supplementary Materials: Anti-Cancer Effect of Quercetin in Xenograft Models with EBV-Associated Human Gastric Carcinoma

Hwan Hee Lee, Seulki Lee, Yu Su Shin, Miyeon Cho, Hyojeung Kang, and Hyosun Cho

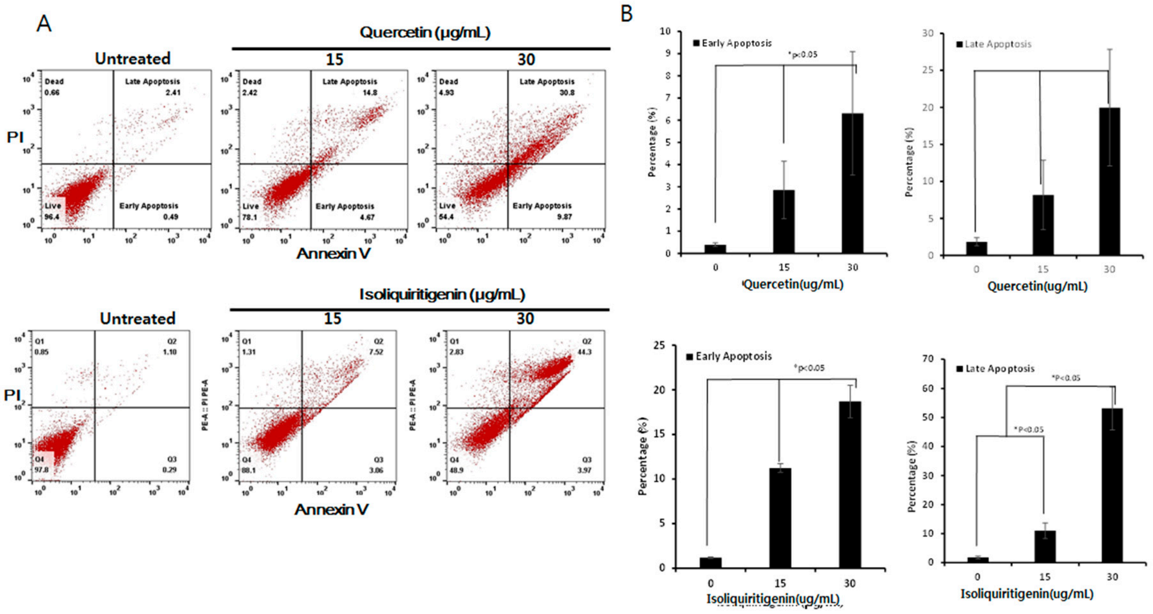

Figure S1. Induction of cell apoptosis by quercetin and isoliquiritigenin in SNU719 cells. Cells were treated with or without quercetin and isoliquiritigenin (15 and 30 μg/mL) for 48 h and then stained with Annexin V and propidium iodide (PI). Apoptosis was analyzed by flow cytometry. (**A**) Representative Annexin V and PI staining for cell apoptosis in SNU719 cells by quercetin (top) and isoliquiritigenin (bottom); (**B**) The percentage of early and late apoptosis are presented as mean ± standard deviation (SD) for three independent experiments.

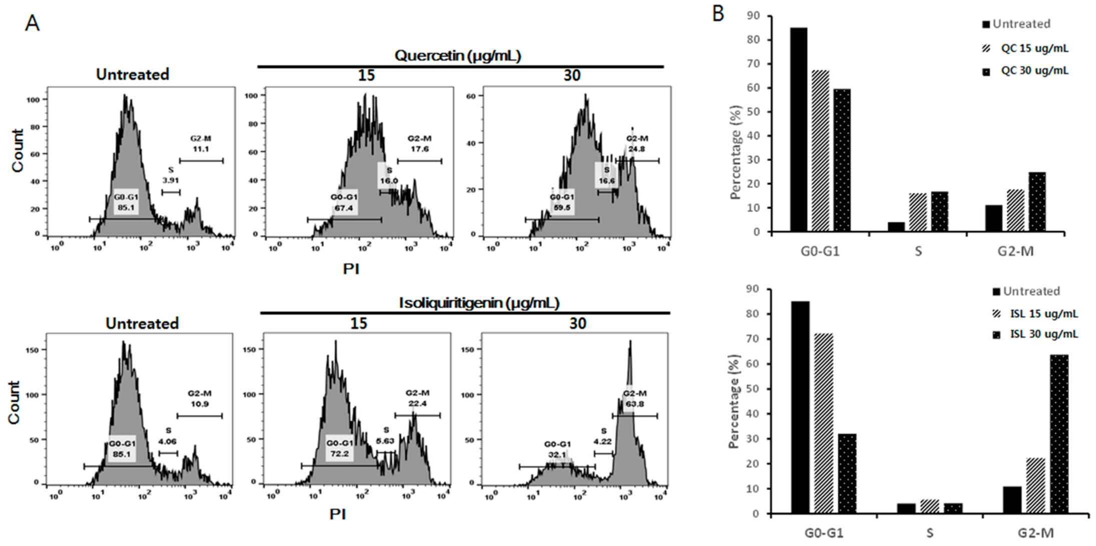

Figure S2. Induction of cell cycle arrest by quercetin and isoliquiritigenin in SNU719 cells. Cells were treated with or without quercetin and isoliquiritigenin (15 and 30 μg/mL) for 48 h. Then, cells were harvested for PI staining, which was measured by flow cytometry. (**A**) Representative PI staining for cell cycle progress in apoptosis in SNU719 cells by quercetin (top) and isoliquiritigenin (bottom); (**B**) The percentage of cell cycle distribution is presented.

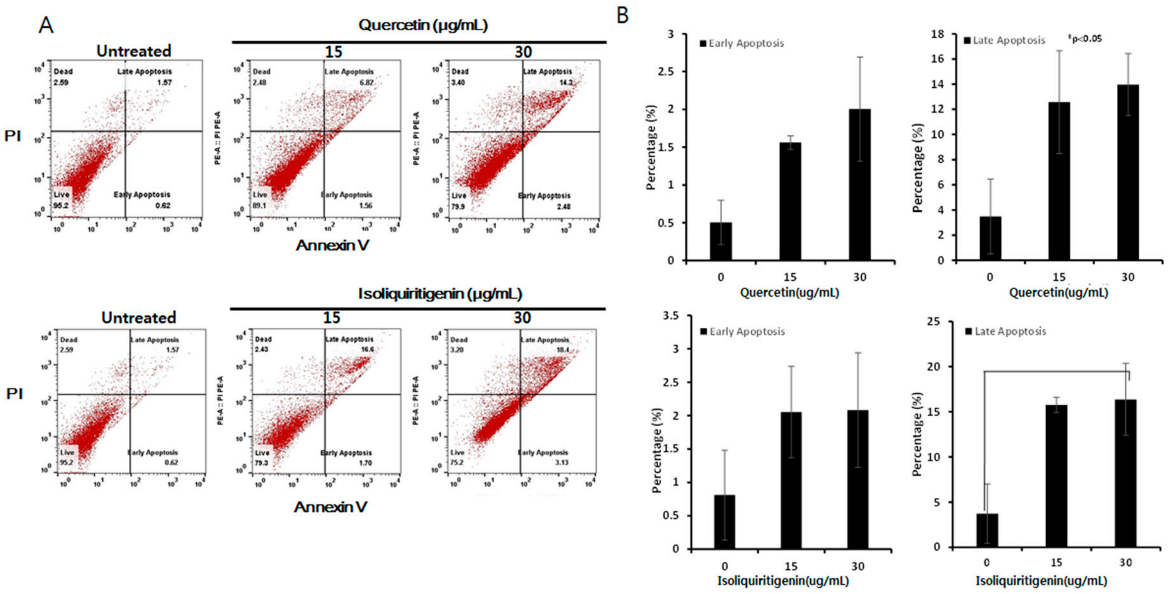

Figure S3. Induction of cell apoptosis by quercetin and isoliquiritigenin in MKN74 cells. Cells were treated with or without quercetin and isoliquiritigenin (15 and 30 µg/mL) for 48 h and then stained with Annexin V and propidium iodide (PI). Apoptosis was analyzed by flow cytometry. (**A**) Representative Annexin V and PI staining for cell apoptosis in MKN74 cells by quercetin (top) and isoliquiritigenin (bottom); (**B**) The percentage of early and late apoptosis are presented as mean ± standard deviation (SD) for three independent experiments.

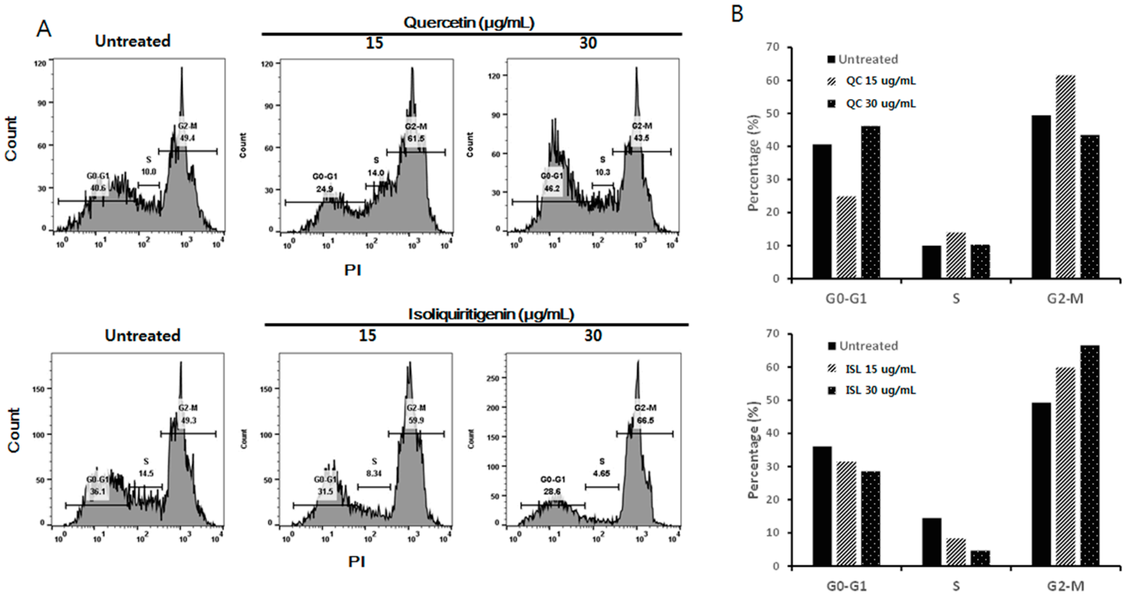

Figure S4. Induction of cell cycle arrest by quercetin and isoliquiritigenin in MKN74 cells. Cells were treated with or without quercetin and isoliquiritigenin (15 and 30 µg/mL) for 48 h. Then, cells were harvested for PI staining, which was measured by flow cytometry. (**A**) Representative PI staining for cell cycle progress in apoptosis in MKN74 cells by quercetin (top) and isoliquiritigenin (bottom); (**B**) The percentage of cell cycle distribution is presented.

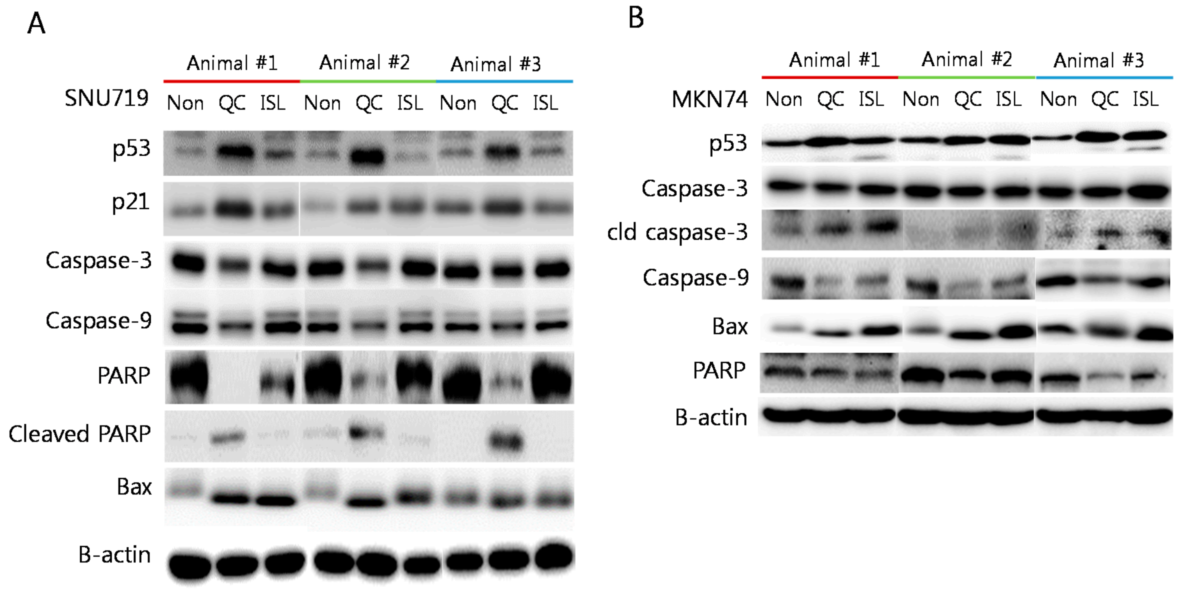

Figure S5. Expressions of p53, p21, (cleaved) caspase-3, -9, Parp and Bax proteins in tumor tissues from mice implanted with EBV(+) (SNU719) or EBV(−)(MKN74) human gastric carcinoma. Tumor tissue was excised from each animal fed with quercetin (QC) or isoliquiritigenin (ISL) (three animals per group) and prepared for western blot analysis. The protein expressions of (**A**) p53, p21, caspase-3, 9 and (cleaved) Parp; (**B**) p53, (cleaved) caspase-3, 9, Bax and Parp were identified and relative intensities were measured. β-Actin was used as the loading control.
